# The effect of psychological factors in pain intensity of patients with chronic pain conditions

**DOI:** 10.1192/j.eurpsy.2023.673

**Published:** 2023-07-19

**Authors:** G. N. Lyrakos, H. U. A. Ogah, E. Aslani, V. Spinaris

**Affiliations:** ^1^Psychiatric, General Hospital Nikaia “Agios Panteleimon”, Nikaia; ^2^Psychology, City Unity College, Athens; ^3^2nd Dep of Pathology, General Hospital Nikaia “Agios Panteleimon”, Nikaia, Greece

## Abstract

**Introduction:**

Chronic pain can lead to depression, weariness, sleep problems, decreased physical and cognitive function, personality changes/shifts, and social interactions, all of which can lead to social marginalization and financial loss.

**Objectives:**

The aim of the present study was to investigate how psychological variables affects pain intensity.

**Methods:**

193 patients diagnosed with chronic pain conditions, men 67 (34.8%) and women 126 (65.2%), participated in the study. This study used a quantitative between-subjects design to investigate the effect of psychological factors on pain intensity using the VAS scale. Analysis was performed with the use of SPSS23.

**Results:**

The analysis produces a coefficient of determination R2 = 0.448 – suggesting that a total 44.8% variability in pain intensity in the previous month can be explained by Age, Fear-avoidance belief about physical activity, Commitment to activity, fear avoidance beliefs about work and Pain catastrophizing magnification. A repeated measure analysis of variance shows that the regression model is statistically significant F (1, 187) = 30.381, p = 0.000. The predictors variables (Age, fear-avoidance belief about physical activity, commitment in activity, fear avoidance beliefs about work and pain catastrophizing magnification) are found to statistically significant t (187) = 9.627, p = 0.001, t (187) = 4.616, p = 0.001, t (187) = 2.982, p = 0.003, t (187) = -2.599, p = 0.010, t (187) = 2.253, p = 0.025 respectively.

**Conclusions:**

The findings of this study are in agreement with previous literature and also provide insight into the major psychological factors correlates with pain intensity

**Disclosure of Interest:**

None Declared

